# The effect of donor human milk on the length of hospital stay in very low birthweight infants: a systematic review and meta-analysis

**DOI:** 10.1186/s13006-020-00332-6

**Published:** 2020-10-28

**Authors:** Rui Yang, Danqi Chen, Qingqi Deng, Xinfen Xu

**Affiliations:** 1 Nursing Faculty, School of Medicine, Zhejiang University, Hangzhou, China; 2 Women’s Hospital, School of Medicine, Zhejiang University, Hangzhou, Zhejiang China; 3 Haining Maternal and Child Health Hospital, Branch of Women’s Hospital, School of Medicine, Zhejiang University, Hangzhou, China

**Keywords:** Donor human milk, Length of hospital stay, Preterm infant

## Abstract

**Background:**

Donor human milk (DHM) is an alternative to preterm infant formula if the mother’s own milk is not available. Since the lactation period and preservation treatment of DHM are different from those of mother’s own milk, we aimed to determine the reduction in the length of hospital stay by DHM compared to preterm infant formula.

**Methods:**

In this systematic review, we searched PubMed/MEDLINE, EMBASE, and the Cochrane Library to retrieve studies on the impact of DHM on the clinical outcomes of preterm infants published before 1 November 2019. The study included very low birthweight (VLBW) infants taking either DHM or infant formula with data on the length of hospital stay. Data were analysed using Review Manager 5.3 software.

**Results:**

The literature search yielded 136 articles, and four randomised controlled trials (RCTs) and eight observational studies met the inclusion criteria. A meta-analysis of the RCTs (*N* = 725) showed no reduction in the length of hospital stay in both the DHM and infant formula groups (− 0.22 days; 95% CI -6.38, 5.95 days), whereas that of the eight observational studies (*N* = 2496) showed a significant reduction in the length of hospital stay in the DHM group (− 11.72 days; 95% CI -22.07, − 1.37 days). A subgroup analysis of the RCTs revealed that the incidence of necrotising enterocolitis (NEC) was significantly lower in the DHM group when the analysis included high-quality RCTs (RR = 0.32; 95% CI 0.15, 0.69).

**Conclusions:**

This systematic review of RCTs showed that DHM neither prolonged nor shortened the length of hospital stay in VLBW infants compared to preterm infant formula; however, it reduced the incidence of NEC, further validating the protective role of DHM in the health and safety of VLBW infants.

## Background

In the past few decades, advances in medical technology and treatment have greatly improved the survival rate of very low birthweight (VLBW) infants; however, they have not reduced the corresponding morbidity [1–3]. Many complications threaten the quality of life of VLBW infants [4]. Neonatal intensive care is associated with high healthcare costs in the economy and society.

Human milk is increasingly recognised for its nutritional and immune effects on neonates, including preterm infants [5]. The American Academy of Pediatrics recommends donor human milk (DHM) as an alternative feeding method if the mother’s own milk is not available [6]. The World Health Organization (WHO), UNICEF, American Academy of Pediatrics, and Japan Pediatric Society suggest that DHM has many advantages compared to preterm infant formula when feeding VLBW infants [7–9]. The European Society for Paediatric Gastroenterology, Hepatology, and Nutrition recommends that preterm infant formula should be used only when DHM and mother’s own milk are not available [10]. There is ample supporting evidence that mother’s own milk diet in preterm infants can reduce the incidence of late-onset sepsis, bronchopulmonary dysplasia (BPD), retinopathy of prematurity (ROP), and necrotising enterocolitis (NEC), as well as improve feeding tolerance, shorten the length of hospital stay, and reduce medical costs [11–13]. DHM and mother’s own milk are not completely equivalent because DHM may be obtained in a different lactation stage and is pasteurised; whether DHM is as good as mother’s own milk when compared to infant formula still needs to be studied. Long hospital stay predisposes VLBW infants to hospital environment-related risks, such as nosocomial infections, noise, and lighting, that may affect development [14, 15]. Studies have shown a positive correlation between shorter hospital stays and better clinical outcomes, lower mortality rates, and fewer readmissions [16, 17]. Similarly, a longer hospital stay can affect the establishment of mother-to-infant or parents-infant attachment [18]. Similarly, the increased hospital stay would affect the allocation of health resources, reducing the number of available Neonatal Intensive Care Unit (NICU) beds and restricting other preterm infants who require hospital care from admission to the NICU. For families with preterm infants, a long hospital stay can cost more, increase visits to hospitals, and cause negative emotions [18–22]. It is not uncommon for a NICU admission to cost more than $3500 per day for a baby, and long-term hospitalisation costs can exceed $1 million [23]. We aimed to conduct a systematic review of randomised controlled trials (RCTs) and observational studies to analyse the impact of DHM on the length of hospital stay in VLBW infants using a meta-analytic approach. The length of hospital stay was regarded as the primary outcome, whereas NEC was the secondary research outcome.

## Methods

We developed a protocol detailing the review question, search strategy, inclusion criteria, data extraction, study quality assessment, and strategy for data synthesis. This protocol has been registered on the International Prospective Register of Systematic Reviews (PROSPERO) with the ID of CRD42019133797 and is available from https://www.crd.york.ac.uk/prospero/display_record.php? ID = CRD42019133797. This article is a systematic review, and the Women’s Hospital School of Medicine Zhejiang University Medical Ethics Committee reviewed it for exemption.

### Search strategies

A literature retrieval was conducted among studies published before 1 November 2019 in PubMed / MEDLINE, EMBASE, and the Cochrane Library. The PubMed search strategy used the following terms, including MeSH terms: (donor milk [Title/Abstract], donor breast milk [Title/Abstract], banked milk [Title/Abstract], “pasteurized human milk” [Title/Abstract], or donor human milk [Title/Abstract]) plus (Infant, Very Low Birth Weight [MeSH]). Using this strategy, the length of hospital stay mostly appeared in the study results rather than the title or abstract; hence, it was not used as one of the search terms. Similar search strategies were used in other databases. No language restrictions were placed on the search results, and translations were made if necessary. RCTs and observational studies evaluating DHM feeding versus formula in VLBW infants were included. Systematic reviews, literature reviews, case studies, qualitative studies, and commentaries were not included but were examined to help identify additional applicable studies.

### Inclusion criteria

Studies meeting the following inclusion criteria were included in the study: 1) study designs involving RCTs and quasi-experimental and observational studies, 2) study population, including very low birthweight infants, with weights < 1500 g or gestational age < 32 weeks, 3) studies grouping subjects according to the feeding protocol, including a DHM group and a infant formula group; we defined the DHM and formula groups as either DHM or formula, respectively, accounting for more than 75% of the total enteral diet, and 4) studies wherein reported outcomes included the length of hospital stay. If several studies targeted the same patients, the study could be included if it met the above inclusion criteria and was published earlier.

### Data extraction

The researchers conducted a preliminary screening of the titles and abstracts of the studies retrieved through the search strategy. Two researchers (RY, DQC) further reviewed the studies and extracted the following information and data: study design, study population, inclusion criteria, primary outcome(s), intervention/groups, length of hospital stay, and incidence of complications. The results of the two researchers’ (RY and DQC) assessments of RCTs were consistent. The interrater-reliability of the observational studies was calculated using SPSS 26.0 (k = 0.826), showing a good agreement between the two researchers. The mean and standard deviation (SD) of continuous variables and the percentage of dichotomous variables in the outcome measure were extracted. We contacted the authors of studies with insufficient data for more detailed information and data. One article [24] was excluded because there was no reply.

### Quality assessment

Risk of bias in RCTs was assessed using the Cochrane Collaboration and the Working Group for Grading of Recommendations Assessment, Development, and Evaluation (GRADE) [25]. RCTs were assessed for the risk of bias through The Cochrane Collaboration’s tool for assessing the risk of bias. Each item was assessed according to the manual, and risks were accordingly categorised as high, moderate, or low. The risk of bias in observational studies was assessed using the Newcastle-Ottawa Scale (NOS) [26] for quality assessment. A star was given if the study matched the description of an item in the NOS manual; studies could receive 1–9 stars (three domains with eight items in total; one item in the comparability section is divided into two). Two investigators independently evaluated the included studies, and studies for which consensus was not reached on quality assessment were evaluated by a third investigator.

### Statistical analysis

Data analysis was performed with the Review Manager 5.3 software. Relative risk (RR) was calculated with a 95% confidence interval (CI) for the dichotomous variable (incidence of NEC). The mean difference (MD) was calculated with 95% CI for continuous variables. To retain valuable studies’ information, studies that provided only the median and interquartile range were included in the analysis. After consulting other meta-analyses that included studies only reporting the median and interquartile range, Wan et al.’s [27] method was applied here to estimate the mean and SD. A study presenting continuous variables as median and range was excluded, and we did not receive usable data from the author. The random-effect model was used to describe the combined effect size. Sensitivity analysis was performed when investigating the effect of individual studies on the combined effect size.

## Results

**Fig. 1:**
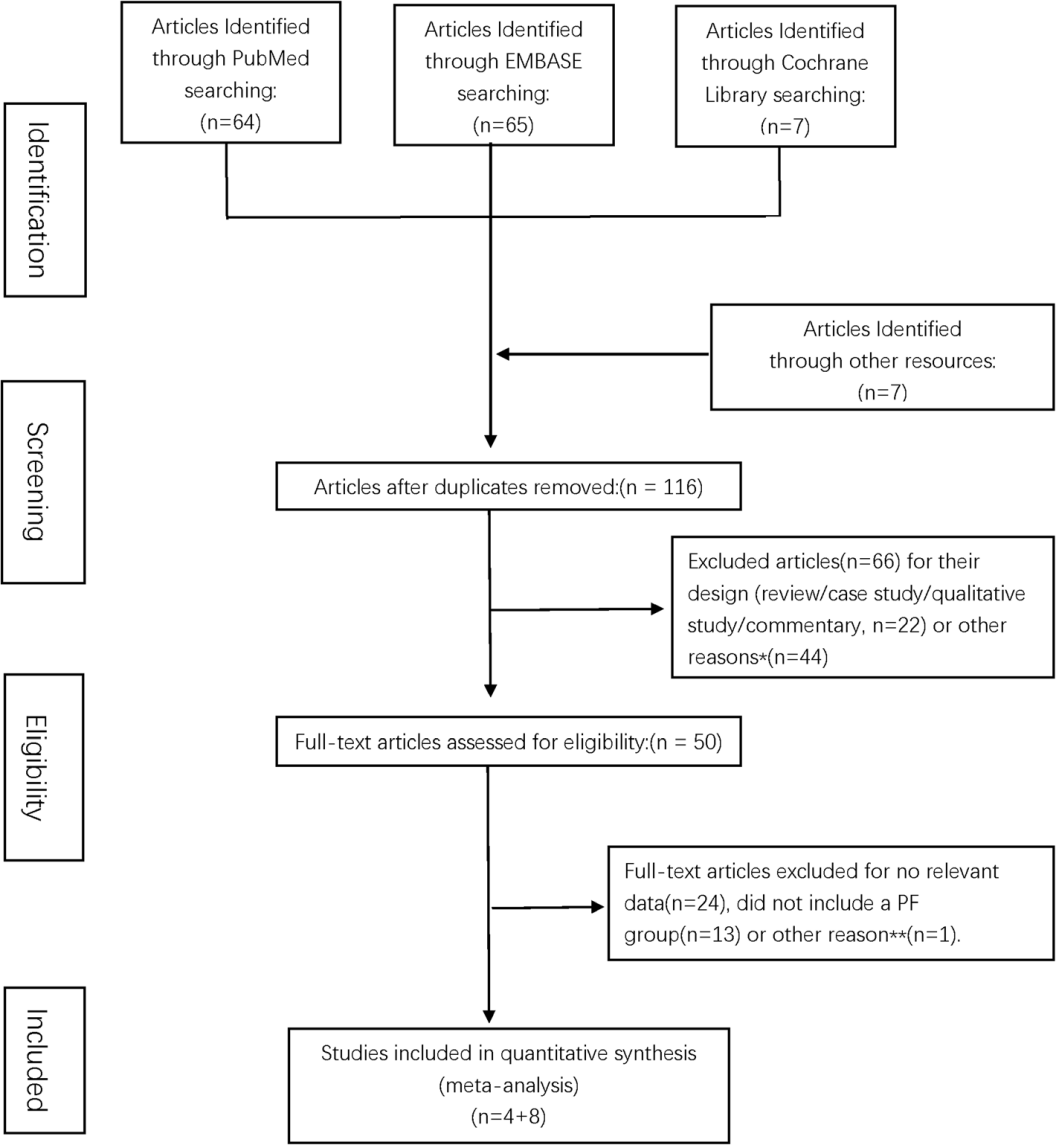
Flow chart of the literature search strategy. *Forty-four studies were excluded as they did not meet the inclusion criteria (study population; studies grouping). **A study was excluded when the continuous variables were presented as median and range, and we did not receive usable data from the author

The literature search per the search strategy yielded 136 articles on completion, and seven potential articles [5, 28–33] were identified by reading reviews or from other resources. Fifty full-text articles were assessed for eligibility after reading the titles and abstracts of 116 articles and removing duplicates. Twenty-four articles were excluded for missing data, such as length of NICU stay, 13 reports were excluded due to the absence of an infant formula group, and a retrospective study [34] was excluded because its data was obtained from one of the RCTs [35] already included. In total, 12 studies, including 4 RCTs [32, 35–37] and 8 observational studies [38–45], that focused on DHM feeding and hospitalisation outcomes in VLBW infants were identified (Fig. 1).

The details of the included studies are shown in Table 1 (RCTs) and Table 2 (observational studies). The four RCTs included 358 VBLW infants fed with DHM and 367 VBLW infants fed with infant formula. Two studies [35, 37] were assessed as having a low risk of bias in all aspects and the other two studies as having high risk due to blinding of participants and/or personnel blinding to outcome assessment following the Cochrane Collaboration’s tool. The eight observational studies reported 1306 VBLW infants fed with DHM and 1190 VBLW infants fed with infant formula. Four of the eight studies were rated 9-star in the assessment of the risk of bias, three studies were rated 7-star for not meeting criteria in the comparability section, and one study was rated 6-star. The details of the study assessments are reported in Table 3 (RCTs) and Table 4 (observational studies).

**Table 1:** Characteristics of included randomized controlled trials

Authors	Year	***n*** of Infants	Design	Inclusion criteria	Gestational age, week	Primary outcome(s)	Intervention	Duration of intervention/observation	Length of hospital stay, days (mean ± SD)	Fortification	Comments
Schanler et al. [37]	2005	243	Single blinded	GA ≤ 29 weeks	27 ± 2/ 27 ± 2	Incidence of late-onset; sepsis and NEC	Exclusively MOM MOM + DHM MOM + PF	90 days or discharge.	85.0 ± 53.0/ 90.0 ± 56.0	Bovine fortifier added to DHM group.	According to established criteria, DHM and PF groups were included.
O’Connor et al. [35]	2016	363	Double blinded	BW < 1500 g, enteral feeding within 7 day	27.5 ± 2.4/ 27.8 ± 2.7	Bayley-III score at 18 month; the incidence of NEC	MOM + DHM MOM + PF	90 days or discharge.	77.2 ± 53.0/ 73.2 ± 39.2	Bovine fortifier added to DHM group.	MOM + DHM group: MOM = 58% MOM + PF group: MOM = 63%
Cristofalo et al. [36]	2013	53	Double blinded	BW < 1250 g	27.7 ± 1.5/ 27.5 ± 2.4	Duration of parenteral nutrition; the incidence of NEC	Exclusive DHM Exclusive PF	91 days of age or discharge, or ≥ 50% oral feedings.	80.0 ± 32.1/ 90.0 ± 50.2	DHM fortifier added to DHM group.	
Sullivan et al. [32]	2010	207	Single blinded	BW 500–1250 g, intention to provide MOM.	27.2 ± 2.2/ 27.3 ± 2.0	The incidence of NEC; parenteral nutrition, days; length of stay, days; bronchopulmonary dysplasia; weight gain	MOM+DHM MOM + PF	91 days or oral feedings or discharge.	80.1 ± 34.8/ 81.3 ± 24.2	Donor added to DHM group; bovine fortifier added to PF group.	Group HM100 and group PF received fortifier when enteral intake was 100 mL/kg/d. The gestational week refers to the two groups rincluded.

*MOM* mother’s own milk, *PF* preterm formula, *DHM* donor human milk, *HM* human milk, *GA* gestational age, *BW* birth weight, *NEC* necrotizing enterocolitis

**Table 2:** Characteristics of included observational studies

Authors	Year	***n*** of Infants	Design	Inclusion criteria	Gestational age, wk	Primary outcome(s)	Groups	Duration of intervention/observation	Length of hospital stay, days (mean ± SD)	Fortification	Comments
Madore et al. [45]	2017	81	Retrospective case-control	BW < 1000 g	27.1 ± 1.9/ 27.3 ± 2.1	Growth; neuro-development; incidence of NEC	Group MOM; Group DHM; Group PF	First month of life	65.5 ± 35.5/86.5 ± 46.6	Bovine fortifier added to MOM and DHM.	According to established criteria, DHM and PF groups were included. Gestational week refers to the two groups included.
Hair et al. [41]	2016	1587	Retrospective cohort	BW < 1250 g	26.5 ± 2.5/ 26.4 ± 2.3	Incidence of NEC; mortality	MOM+ DHM + DHM-based fortifier; MOM+ PF + bovine fortifier	32–34 corrected GA, or 60 days of life.	92.4 ± 54.5/94.7 ± 62.1	DHM-based fortifier added to DHM group. Bovine fortifier added to PF group.	
Ginovart et al. [40]	2016	186	Retrospective cohort	BW < 1500 g	29.1 ± 2.9/ 29.6 ± 2.9	Retinopathy of prematurity; incidence of NEC	MOM+ DHM; MOM + PF	Four weeks	65.0 ± 34.0/58.5 ± 32.0	Bovine fortifier added to MOM and DHM.	
Lee et al. [44]	2016	46	Retrospective cohort	BW < 1500 g	27.8 ± 1.4/ 28.0 ± 1.7	Morbidity, duration of parenteral nutrition	MOM + DHM; MOM + PF	Not specified.	61.1 ± 17.8/74.3 ± 21.5	Not specified.	
Kreissl et al. [43]	2017	283	Prospective study	BW < 1500 g; GW < 32 w	27.6 (23.3–31.9)/ 27.3 (23.3–21.3) [Median (range)]	Full enteral feeding; weight at discharge; incidence of NEC	Group DHM; Group PF	Not specified.	60.0 ± 38.2/94.0 ± 37.5	Bovine fortifier added to MOM and DHM	
Kim et al. [42]	2017	90	Retrospective cohort	BW < 1500 g; GW < 32w	32.3 ± 4.3/ 31.0 ± 3.9	In-hospital mortality; broncho-pulmonary dysplasia; early-onset morbidity; growth parameters; incidence of NEC	Group DHM; Group PF	Not specified.	60.5 ± 19.9/75.5 ± 28.3	HM fortifier added to DHM group	
Colaizy et al. [39]	2012	171	Retrospective cohort	BW ≤ 1250 g; GA < 27 w	27 (25.6, 28.8)/ 28.43 (25.4, 29.6) [median (IQR)]	Growth; morbidity; incidence of NEC	< 25%HM + formula; 25–50%HM + formula; 51–75% HM + formula; > 75% HM + formula	Not specified	77.3 ± 24.8/83.7 ± 54.9	Bovine-based fortifier added to > 75% DHM group, which is a subgroup of human milk	< 25% HM + formula and > 75% DHM groups were included. Gestational week refers to the two groups included.
Chowning et al. [38]	2016	550	Retrospective cohort	BW ⩽1500 g	29.1 ± 2.4/ 29.3 ± 2.8	Mortality rate; incidence of NEC surgical; NEC rate	0% HM; < 50% DHM; ⩾ 50% DHM; ⩾90D HM	Not specified.	56.5 ± 22.0/65.9 ± 60.2	Bovine-based fortifier added to ⩾90 HM group	According to established criteria, 0% HM and ⩾90 DHM groups were included. Gestational week refers to the two groups included.

*MOM* mother’s own milk, *PF* preterm formula, *DHM* donor human milk, *HM* human milk, *GA* gestational age, *BW* birth weight, *NEC* necrotizing enterocolitis

**Table 3:** Assessment of risk of bias of included randomized controlled trials^a^

	Random sequence generation	Allocation concealment	Blinding of participants and personnel	Blinding of outcome assessment	Incomplete outcome data	Selective reporting	Other bias	Total
O’Connor et al. [35]	A	A	A	A	A	A	A	A
Sullivan et al. [32]	A	A	C	A	A	A	A	C
Schanler et al. [37]	A	A	C	C	A	A	A	C
Cristofalo et al. [36]	A	A	A	A	A	A	A	A

^a^ A, high risk; B, moderate risk; C, low risk

**Table 4:** Assessment of the quality of included observational studies^a^

	Selection	Comparability	Outcome	Total
Lee et al. [44]	4	0	2	6
Kreissl et al. [43]	4	0	3	7
Kim et al. [42]	4	2	3	9
Colaizy et al. [39]	4	2	3	9
Chowning et al. [38]	4	2	3	9
Hair et al. [41]	4	0	3	7
Madore et al. [45]	4	2	3	9
Ginovart et al. [40]	4	0	3	7

^a^ Numbers represent the total number of stars obtained by the observational study in each quality domain

The publication bias of the RCTs included was invisibility on a funnel plot due to the limited number of studies. No evidence of publication bias was found in the analysis. The observational studies did not reveal publication bias through the use of the Egger’s test (*p* = 0.097).

### Effects on length of hospital stay

**Fig. 2:**
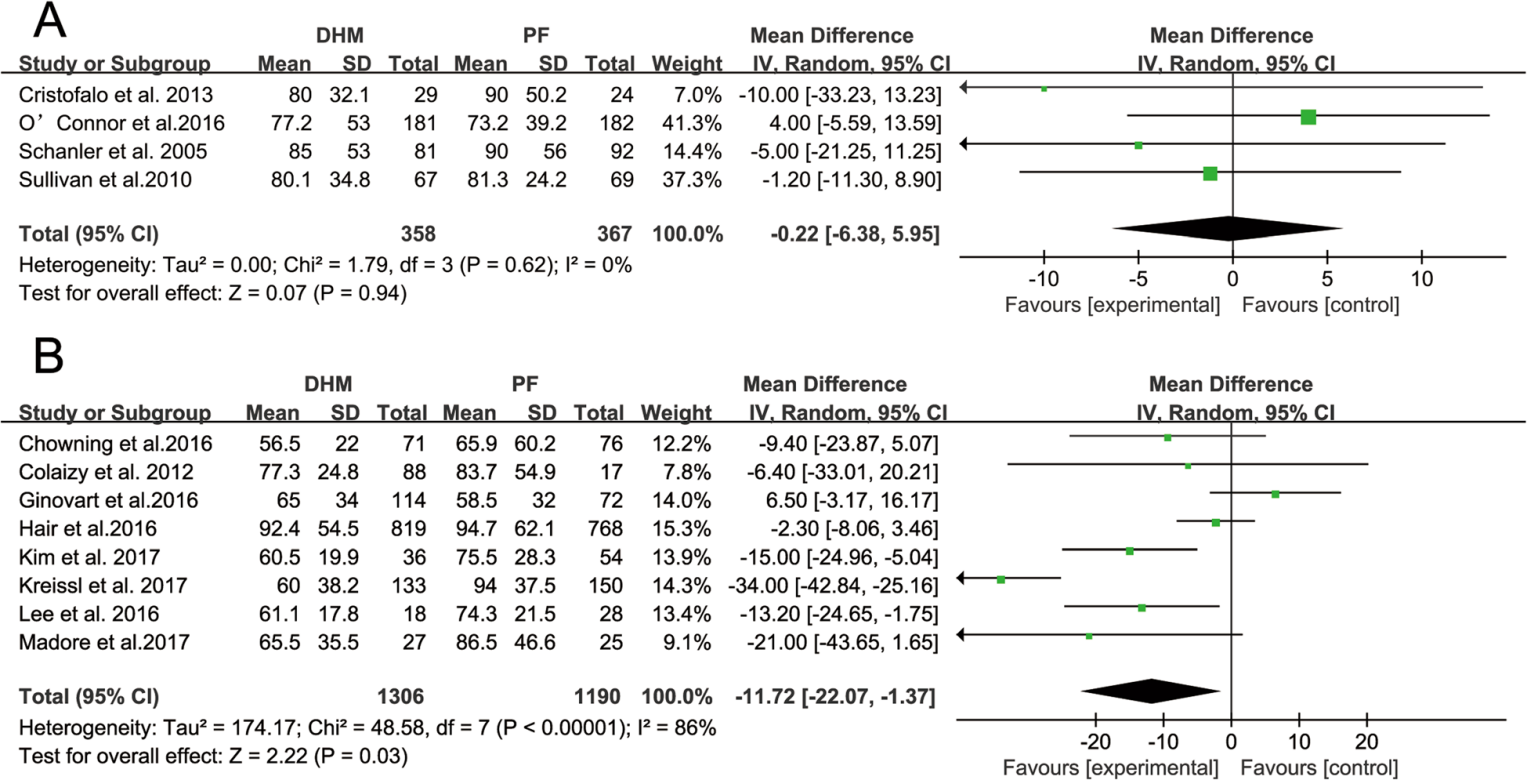
The effects of DHM on the length of hospital stay compared to PF. A. RCTS; B. observational studies. Squares (■) represent the effect size of each study. Diamonds (◆) represent the pooled effect size. DHM, donor human milk; PF, preterm infant formula

The length of hospital stay of 725 infants from four RCT studies was measured. The meta-analysis did not demonstrate a significant reduction in length of hospital stay in the DHM group compared to the infant formula group (− 0.22 days; 95% CI -6.38, 5.95 days; *p* = 0.94). The heterogeneity was not significant (I^2^ = 0%, *p* = 0.62). Eight observational studies with 2496 infants were included in the meta-analysis, and a significant reduction in length of hospital stay for infants receiving DHM (− 11.72 days; 95% CI -22.07,-1.37 days; *p* = 0.03) was observed. The heterogeneity was statistically significant (I^2^ = 86%, *p* = 0.03). The forest plot of the effect is presented in Fig. 2.

### Effects on NEC

**Fig. 3:**
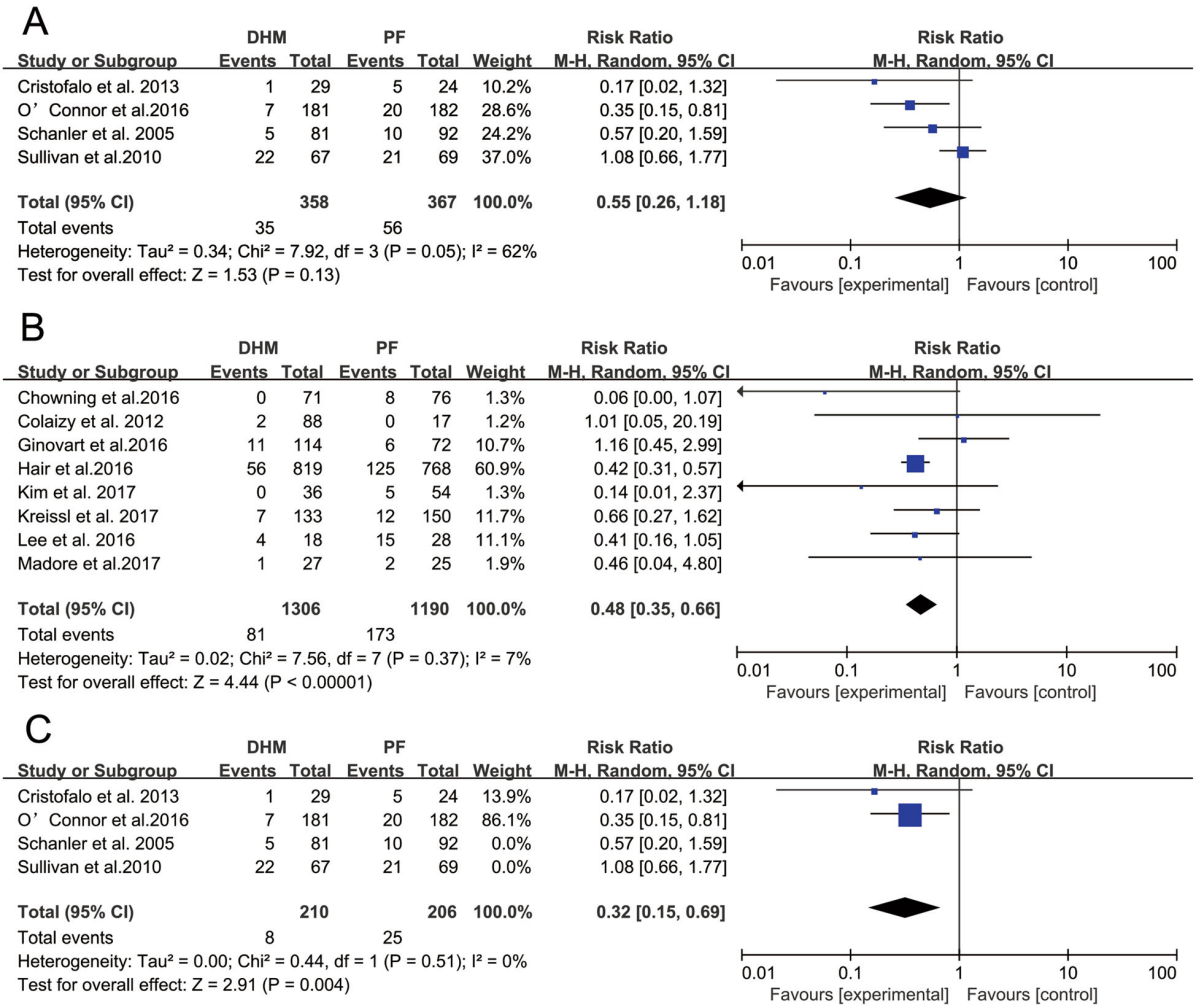
The effects of DHM on NEC compared to PF. A. RCTS; B. observational studies; C. high-quality RCTs studies. Squares (■) represent the effect size of each study. Diamonds (◆) represent the pooled effect size. DHM, donor human milk; PF, preterm infant formula

Four RCT studies with a total sample of 725 infants measured the occurrence rates of infant NEC. The meta-analysis did not find a significantly lower risk ratio for NEC in the DHM group compared with the control group (RR = 0.55; 95% CI 0.26, 1.18; *p* = 0.13). The heterogeneity was statistically significant (I^2^ = 62%, *p* = 0.05). Eight observational studies with 2496 infants were included in the meta-analysis, and a significant reduction in the occurrence of NEC was observed in infants receiving DHM (RR = 0.48; 95% CI 0.35, 0.66; *p* < 0.05). The heterogeneity was not statistically significant (I^2^ = 7%, *p* = 0.37), and the forest plot of the effect is presented in Fig. 3.

A subgroup analysis (Fig. 3c) of RCTs indicated that the effect of DHM on NEC was statistically significant in reducing the incidence of NEC when the subgroup analysis was restricted to high-quality RCTs (RR = 0.32; 95% CI 0.15, 0.69; *p* = 0.004), and the heterogeneity was not significant (I^2^ = 0%, *p* = 0.51).

## Discussion

From the meta-analysis of the pooled RCTs data, DHM was not observed to affect the length of hospital stay of VLBW infants. There was no evidence that DHM lowered the incidence of NEC in VLBW infants; however, a meta-analysis of high-quality RCTs did reveal that DHM significantly reduced the incidence of NEC, whereas that of observational studies (eight studies) showed an improvement in length of hospital stay in the DHM group when compared to the infant formula group. Additionally, a meta-analysis of observational studies (eight studies) also revealed that feeding infants with DHM compared to infant formula reduced the incidence of major medical complications, such as NEC.

Length of hospital stay is a complex measure that has many interfering factors. Multiple factors, other than nutritional intake, have been identified as confounding variables associated with infant development and may even manifest before birth [46]. For VLBW infants, the difference in birth weight of 100 g or 1 week of gestational age can have a significant impact on perinatal and postnatal complications. These complications have a direct or indirect effect on the length of hospital stay in these infants and are likely to confound the influences of DHM [47].

DHM may influence the length of hospital stay as follows: (a) by improving the nutrition and growth of infants; (b) by reducing the incidence of late-onset sepsis in VLBW [48, 49]; and (c) through critical nutrients, such as long-chain polyunsaturated fatty acids (LCPUFA), and possibly other neurotrophic factors of human milk.

One randomised, blinded trial on the feeding of extremely preterm infants included in this analysis concluded that DHM offered a minute short-term advantage over infant formula and mother’s own milk and was associated with fewer infection-related events and shorter hospital stay [37]. The study by O’Connor et al. [35] was a pragmatic, double-blind, randomised trial conducted in four tertiary care NICUs. Similarly, this study found no advantage of feeding VLBW infants with DHM compared to formula at a corrected age of 18 months, as assessed by the Bayley-III. DHM and mother’s own milk differ in their bioactive components, such as live cells and lactoferrin, that play an important role in reducing morbidity (e.g. sepsis). This is due to the process of pasteurisation, which in turn affects neurodevelopment, and might be the reason why the hypothesised improvement associated with DHM was not observed.

Studies have shown that DHM may differ depending on the specific circumstances of the donor mother and the process of pasteurisation [50–52]. The composition of breast milk is related to the mother’s dietary habits, environment, and lactation period. Pasteurisation eliminates immune cells in human milk but does not completely obliterate biological activity, with many bioactive components preserved, including cytokines and growth factors [53]. The timing, volume, and duration of human milk may have an impact on the development of infants. The advantages of human milk in the neurodevelopment of infants are not only reflected during hospitalisation, but after discharge as well [54]. Breastfeeding is an unmatched way of providing ideal food for the healthy growth and development of infants. WHO actively promotes human milk as the best source of nourishment for infants [8]. More research is needed to investigate whether DHM is a priority choice for infants when the mother’s own milk is unavailable.

The included RCTs did not find beneficial short-term outcomes in feeding DHM as a substitution to extremely preterm infants; however, long-term outcomes of infants fed with DHM compared to infant formula need to be investigated. As the use of DHM increases, due to the differences between DHM and mother’s own milk, the DHM processing and DHM protocol in the NICU will require high-quality clinical assessments.

The present study has the following limitations: 1) the number of included RCTs is small; 2) none of the RCTs were initially designed to investigate the effect of DHM on the length of hospital stay in preterm infants; 3) the included RCTs were conducted over a specific period, which may introduce bias; and 4) fortification strategies among DHM studies were different, especially regarding the type of fortification (bovine vs. human milk). Despite these limitations, this systematic review revealed that there are benefits in feeding VLBW infants with DHM compared to infant formula.

## Conclusions

This systematic review of RCTs showed that DHM neither prolonged nor shortened the length of hospital stay in VLBW infants compared to infant formula; however, it reduced the incidence of NEC. Our results are consistent with previously published reviews focusing on the effects of DHM on NEC [11, 55, 56]. Although observational studies have certain methodological limitations, the data suggest that DHM can shorten the length of hospital stay and reduce the incidence of NEC in VLBW infants, further proving the protective effect of DHM on the health and safety of VLBW. Therefore, health facilities should consider improving the application of DHM for VLBW. Similarly, human milk banks should increase publicity on human milk donations and establish increased DHM’s reserves to prepare for its widespread application.

## Data Availability

Not applicable.

## Abbreviations

BPD: Bronchopulmonary dysplasia
DHM: Donor human milk
GRADE: Grading of recommendations assessment, development, and evaluation
LCPUFA: Long-chain polyunsaturated fatty acids
MD: Mean difference
NEC: Necrotising enterocolitis
NICU: Neonatal Intensive Care Unit
NOS: Newcastle-Ottawa Scale
RCTs: Randomised controlled trials
ROP: Retinopathy of prematurity
SD: Standard deviation
VLBW: Very low birth weight
WHO: World Health Organization
